# Dissociation between face perception and face memory in adults, but not children, with developmental prosopagnosia

**DOI:** 10.1016/j.dcn.2014.07.003

**Published:** 2014-08-01

**Authors:** Kirsten A. Dalrymple, Lúcia Garrido, Brad Duchaine

**Affiliations:** a Institute of Child Development, University of Minnesota, Minneapolis, USA; b Department of Psychology, Brunel University, London, UK; c Department of Psychological and Brain Sciences, Dartmouth College, Hanover, USA

**Keywords:** Children, Development, Developmental prosopagnosia, Face memory, Face perception, Face recognition

## Abstract

Cognitive models propose that face recognition is accomplished through a series of discrete stages, including perceptual representation of facial structure, and encoding and retrieval of facial information. This implies that impaired face recognition can result from failures of face perception, face memory, or both. Studies of acquired prosopagnosia, autism spectrum disorders, and the development of normal face recognition support the idea that face perception and face memory are distinct processes, yet this distinction has received little attention in developmental prosopagnosia (DP). To address this issue, we tested the face perception and face memory of children and adults with DP. By definition, face memory is impaired in DP, so memory deficits were present in all participants. However, we found that all children, but only half of the adults had impaired face perception. Thus, results from adults indicate that face perception and face memory are dissociable, while the results from children provide no evidence for this division. Importantly, our findings raise the possibility that DP is qualitatively different in childhood versus adulthood. We discuss theoretical explanations for this developmental pattern and conclude that longitudinal studies are necessary to better understand the developmental trajectory of face perception and face memory deficits in DP.

## Highlights

• Individuals with developmental prosopagnosia (DP) have impaired face recognition.
• In theory, DP could involve impaired face memory, impaired face perception, or both.
• Memory deficits were present in all of our child and adult DPs.
• All children, but less than half of the adults had impaired face perception.

Prosopagnosia is a neurocognitive disorder characterized by severely impaired face recognition (Bodamer, 1947). Individuals with prosopagnosia fail to recognize familiar faces, such as those of family or friends, and sometimes even their own face in the mirror or in photographs. Acquired prosopagnosia results from damage to one or more parts of the face processing system, while developmental prosopagnosia (sometimes called congenital prosopagnosia) results from a failure to develop the mechanisms necessary for face recognition (Behrmann and Avidan, 2005, Susilo and Duchaine, 2013).

Although prosopagnosia is defined as a disorder of face *recognition*, models of face processing decompose recognition into discrete cognitive stages. For example, Bruce and Young's (1986) influential model hypothesizes a separation between structural encoding of a face and face recognition units, which encode face memories. This division suggests that impaired face recognition can result from failures at one or more stages. More recent neurocognitive models of face processing also distinguish between face recognition processes involved in the visual analysis of faces, and those involved in facial familiarity (Gobbini and Haxby, 2007, Haxby et al., 2000). These neurocognitive models use functional imaging data to link these stages to distinct neuroanatomical regions in occipito-temporal cortex, and predict that a failure to develop these units or damage to them would result in particular types of face processing deficits.

Findings from a variety of sources shed light on the relationship between face perception and face memory. Here we define face perception as a set of processes that allow us to represent the properties of a face (with minimal memory demands), and face memory as a set of processes that allow us to store, retain, and later retrieve facial identity information. Behavioral data from individuals with acquired prosopagnosia has supported a division between face perception and face memory. Although some acquired cases are impaired at tests of face perception *and* face memory (e.g. Barton et al., 2004, Busigny et al., 2014, Dalrymple et al., 2011), others demonstrate normal accuracy for face perception (though some have slower than normal reaction times) (Barton et al., 2004, Dalrymple et al., 2011, Tippett et al., 2000).^1^ It has been proposed that prosopagnosia with perceptual deficits results from occipitotemporal lesions, while prosopagnosia in the absence of perceptual deficits results from more anterior lesions (Barton, 2008, Barton and Cherkasova, 2003, Barton et al., 2002, Damasio et al., 1990, Davies-Thompson et al., 2014), though Busigny et al.’s (2014) recent report suggests anterior lesions can also disrupt perception.

Research on the normal development of face perception has also suggested a distinction between face perception and face memory. Face perception appears to mature early, and at the same rate as perception for other objects, while face memory develops more slowly, over the first ten or more years of life, and with a more protracted developmental trajectory than memory for other classes of objects (Weigelt et al., 2014). Data from atypical development also speak to this dissociation. A recent review on face processing in autism spectrum disorders (ASD) suggests that apparent discrepancies in findings of normal versus abnormal face processing in ASD can be explained by the dissociation between face perception and face memory (Weigelt et al., 2012). After an analysis of 90 studies of face processing in ASD, Weigelt et al. (2012) concluded that participants with ASD exhibit face processing impairments when tasks include a memory demand, even if the demand is minimal (e.g. in a sequential matching task with short delay) whereas most tasks of face perception did not reveal face processing deficits.

The dissociation between face perception and face memory has received little attention in the context of developmental prosopagnosia (DP) (Bowles et al., 2009, Stollhoff et al., 2011). Many cases of DP have been reported in detail, and while a number of these cases are impaired at both face perception and face memory (e.g. Chatterjee and Nakayama, 2012, Duchaine et al., 2007a, Duchaine and Nakayama, 2006b, Duchaine et al., 2007b, Nunn et al., 2001, Palermo et al., 2011, Yovel and Duchaine, 2006), some cases achieve normal scores on tests of facial identity perception (Behrmann et al., 2005, Chatterjee and Nakayama, 2012, Humphreys et al., 2007, McKone et al., 2011, Palermo et al., 2011). However, response times are not always provided, leaving the possibility that what appears to be normal performance may instead be the application of successful, but abnormal, feature matching strategies (Busigny et al., 2014, Duchaine and Nakayama, 2004, Farah, 2004, Newcombe, 1979). In support of this suggestion, the reports that did include reaction time (i.e. Behrmann et al., 2005, Humphreys et al., 2007) indicate that the DPs were significantly slower at the perceptual tasks than controls.

Thus it remains unclear whether face perception and face memory are dissociable in DP. Determining whether this dissociation exists in children and adults could illuminate the development and organization of face processing as well as the developmental trajectory of DP. Very little work has been done to characterize DP in children (Dalrymple et al., 2012). The largest sample size of child DPs to-date is three (Wilson et al., 2010), and the remaining studies each report only a single case (Ariel and Sadeh, 1996, Brunsdon et al., 2006, de Haan and Campbell, 1991, Jones and Tranel, 2001, Joy and Brunsdon, 2002, McConachie, 1976, Schmalzl et al., 2008). Yet testing face memory and face perception in children with DP is particularly important: children may be less adept than adults at using compensatory strategies for recognizing faces because they have had less time to develop such strategies in daily life, and they may be less likely to devise alternative strategies that are effective in laboratory tests. Understanding DP in children is also of critical importance given the psychosocial impact of DP on these children and their families (Dalrymple et al., 2014). Accordingly, we tested the face perception and face memory abilities of children with DP to determine whether some individuals show evidence of preserved face perception despite impairments of face memory. We also report comparable data from adults with DP to determine whether qualitatively similar patterns of face recognition deficits are present in children and adults. At an individual level, knowing if particular individual is impaired with face perception, face memory, or both, will have important implications for the design of condition-specific interventions.

## 1 Study 1: Children

### 1.1 Method

#### 1.1.1 Participants

Potential participants were selected from a group of children whose parents reported that their child experiences face recognition difficulties. These parents contacted us through our website faceblind.org or by email. Families who expressed an interest in participating in research studies completed a preliminary screening questionnaire, which was used to determine whether the children met our inclusion criteria. The primary criteria were that children were at least 5-years-of-age, had normal or corrected-to-normal vision, no history of brain trauma, and no diagnosis of autism or Asperger's syndrome.

The parents of children who met our inclusion criteria were contacted by email to ask if they were interested in having their child complete an in-home assessment of face recognition (one child participated in the lab). A member of the research team (KAD) traveled to the family homes. Eight children with DP were identified (3 females) and were included in the study. The mean age of these children was 8.5 years (SD = 2.6, range 5–12). All but one child (OP) were right handed. Parents and children first signed permission and assent forms to confirm their willingness to volunteer in the study. Assessment took one day, and children were compensated for their participation at the end of the day. Information about control participants is included with the test descriptions (below). This study was approved by the Committee for the Protection of Human Subjects at Dartmouth College.

#### 1.1.2 Assessment

Two tests of face memory (Cambridge Face Memory Test-Kids, Old/New Faces) were used to confirm prosopagnosia in the children with suspected DP. These children were additionally assessed with one test of face perception (Dartmouth Face Perception Test). Tests are described below. To determine whether impaired scores on face tests may have resulted from general factors (e.g., poor test-taking skills, lack of interest), we evaluated IQ (Wechsler Abbreviated Scale of Intelligence-II, Wechsler, 2011) and contrasted face memory with memory for other objects. Object memory was assessed with tests that were matched to the face memory tests in terms of format and difficulty. We also assessed low-level vision using the length, size, orientation, and position of gap subscales of the Birmingham Object Recognition Battery (BORB, Riddoch and Humphreys, 1993). OP was unavailable for IQ testing or the BORB, and completed one of the two object memory tests. SWJ did not complete the BORB. BORB performance from the remaining children was compared to the published norms from adults that are distributed with the test (Riddoch and Humphreys, 1993). All BORB scores were in the normal range except CN was in the impaired range on the position of gap subscale. We believe this single impaired score is not sufficient to suggest low-level visual impairments, because CN's object memory score was above average.

**Fig. 1:**
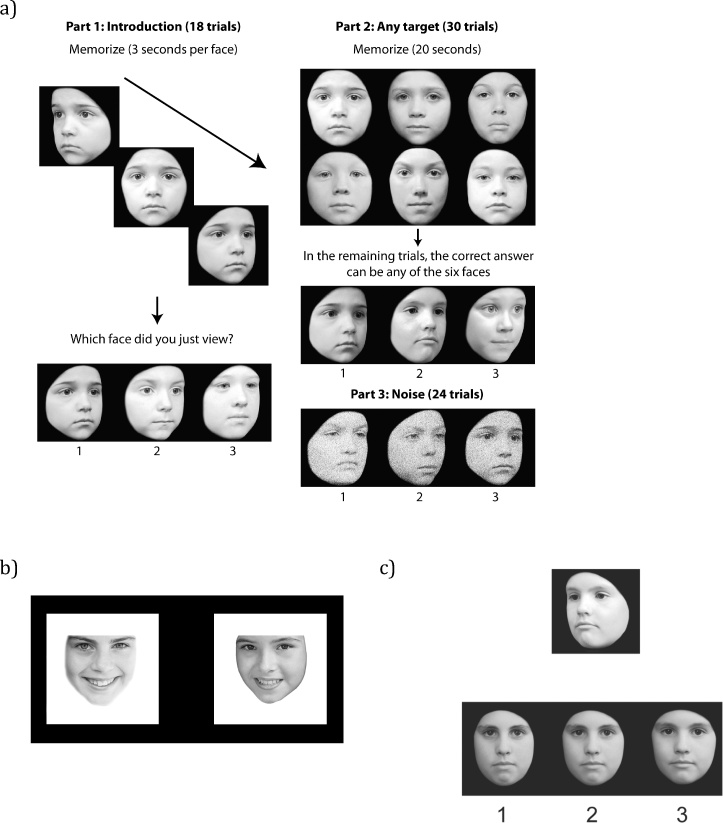
Examples from children's tasks: (a) Cambridge Face Memory Test-Kids (CFMT-K), (b) Old/New Faces, and (c) Dartmouth Face Perception Test (DFPT). CFMT-K instructions appear in the figure. Correct choices in this example are 1, 1, and 3. The Old/New test requires that participants identify which face is one of the 10 target faces memorized in Part 1 of the task. The DFPT requires participants to identify which of the three choice faces looks the most like the target face at the top of the screen. Choice faces in the DFPT are selected from a morph continuum between the target face and another face. The correct choice in this example is 3.

**Fig. 2:**
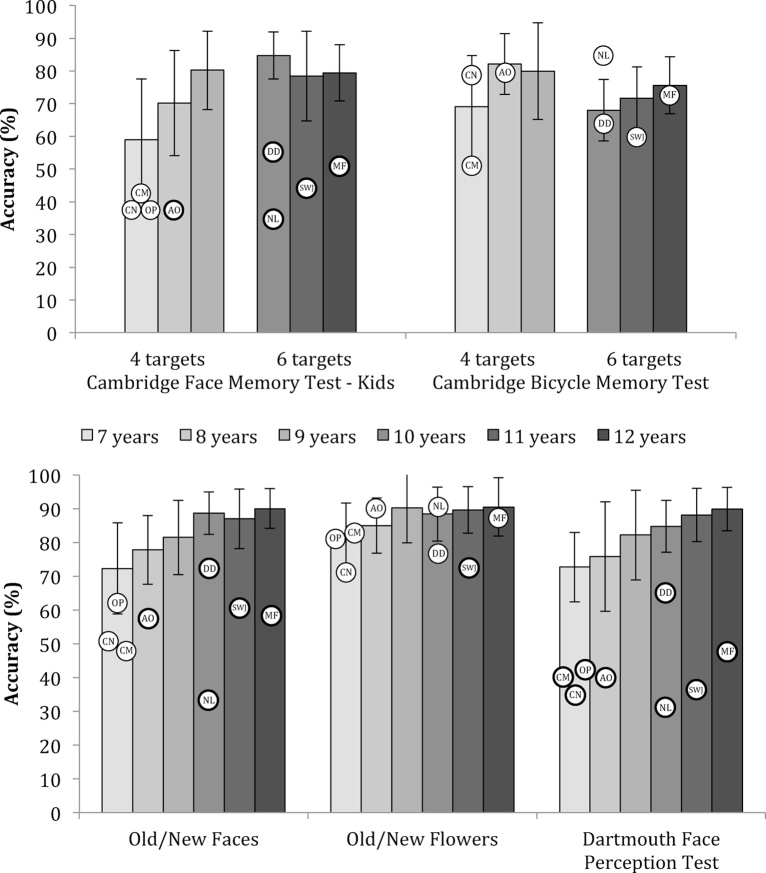
Scores for control participants and DP participants. Data from typically developing children on Cambridge Face Memory Test-Kids (CFMT-K), Cambridge Bicycle Memory Test (CBMT), Old/New Faces, Old/New Flowers, and the Dartmouth Face Perception Test. For the CFMT-K and CBMT 7–9-year-olds memorized 4 targets and 10–12-year-olds memorized 6 targets. Chance level performance on the CFMT-K, CBMT, and DFPT is 33%; chance on the Old/New tasks is 50%. Circles indicate scores from children with DP; letters identify individual participants. Circles with thick lines indicate scores that were >2 SD below the mean. Error bars indicate standard deviation.

Below are descriptions of the two tests of face memory, and the test of face perception used with the children with DP. Example trials from the tests are in Fig. 1. For each test, the data from each child with DP were compared to data from between 12 and 20 typically developing children of the same age (CN, CM, and OP were compared to 7-year-olds). Object memory tasks were identical to the face memory tasks except that the stimuli were bicycles (matched in format to CFMT-K) or flowers (matched in format to Old/New Faces) instead of faces. It is challenging to match the difficulty of face and non-face tasks across all ages because of differences in the rate of development between face and non-face memory (Weigelt et al., 2014), but as can be seen in Supplementary Table 1, the 4-target versions of the CFMT-K and CBMT were particularly well matched in difficulty for 9-year-olds, the 6-target versions of these tasks were particularly well matched for 12-year-olds, and the Old/New Faces and Old/New Flowers were particularly well matched for 10-, 11-, and 12-year-olds. Fig. 2 shows accuracy means and standard deviations from typically developing children, with scores from DPs overlaid. Raw scores are provided in Supplementary Tables 1 and 2.

Supplementary material related to this article can be found in the online version at http://dx.doi.org/10.1016/j.dcn.2014.07.003.

Table S1 Mean scores (%) by age for typically developing children on tests of face memory, face perception, and object memory.

Table S2 Accuracy, and modified *t*-statistics (Crawford and Garthwaite, 2002, Crawford and Howell, 1998) for children with developmental prosopagnosia on subscales of the Birmingham Object Recognition Battery (BORB, Riddoch and Humphreys, 1993), tests of face and object memory, and face perception.

##### 1.1.2.1 Face memory tests

###### 1.1.2.1.1 Cambridge Face Memory Test-Kids (CFMT-K)

The Cambridge Face Memory Test-Kids is a memory task based on the adult version of the task (CFMT, Duchaine and Nakayama, 2006a). Unlike the original CFMT, the CFMT-K uses faces of children instead of adults. Targets and distractors are male faces with neutral expressions chosen from the Dartmouth Database of Children's Faces (Dalrymple et al., 2013) and cropped so that hair and ears were removed.

This task begins with a practice session. A cartoon face is presented three times from three different angles (30° left, front, 30° right) for 3 s each. The participant is asked to try to remember the face and then to pick it out from a choice of three cartoon faces. Choice faces are presented at 30° left, front, 30° right, on three separate trials. The practice session is designed to familiarize the participant with the format of the test.

In the first part of the test the participant is introduced to the target faces using a procedure identical to the practice session, except that real faces are used instead of cartoons. Children 10-years-of-age and older learn six target faces (18 trials in Part 1), and children 9-years-of-age and younger learn four targets (12 trials in Part 1). In the second part of the test the participant is asked to review frontal views of the target faces, which are presented together on the screen for 20 s. At the end of the review period, test trials again consist of three choice faces. The participant is told that one of the choice faces is one of the targets, but is not informed which target will appear on any given trial. Each target appears five times in the second part of the test (6 targets: 30 trials; 4 targets: 20 trials). In the final part of the task, the participant is again asked to review the target faces for 20 s and then to choose the targets from a choice of three faces. This final part of the task differs from the second part because visual noise is added to the choice faces. Each target appears four times (6 targets: 24 trials; 4 targets: 16 trials). In total, the children 10-years-of-age and older children complete 72 trials, while children 9-years-of-age and younger complete 48 trials. Testing takes 10 to 15 min. Chance level performance on these tasks is 33.3%. Data from 92 typically developing children between 7 and 12 years indicates that the six-target version of this test has good internal consistency (Cronbach's *α* = 0.89), and data from a separate group of 55 typically developing children between 7 and 9 years showed the four-target version of this test has comparable internal consistency (Cronbach's *α* = 0.89).

*Old/New Faces.* Ten target and 30 distractor faces were chosen from the Internet. All faces were female children, and were matched for age, facial orientation, and facial expression. Faces were grayscale and hair, ears, and any identifiable moles or freckles were removed.

For the encoding portion of this task, target faces are presented one at a time for 3 s each in the center of the screen. Targets are immediately shown again for 3 s each, and in the same order (i.e. each target was presented twice). The participant is instructed to look at the faces and try to remember them. For the test phase, one target and a similar-looking distractor appear simultaneously on the screen for 1 s. The participant is asked to press a key to indicate which face is one of the target faces (i.e. which is the “old” face). If the participant does not respond within the 1 s window, a blank screen with text, “Please respond now” appears, which remains until a response is provided. Targets appear three times each in random order, for a total of 30 trials. There are 30 unique distractors, and distractors are never repeated. Chance level performance for this test is 50%. Data from a group of 93 typically developing children between 7 and 12 years indicates that this test has acceptable internal consistency (Cronbach's *α* = 0.68).

##### 1.1.2.2 Face perception test

###### 1.1.2.2.1 Dartmouth Face Perception Test (DFPT)

This test begins with three practice trials. In these trials, a cartoon face is presented at the top of the screen facing 30° to the viewer's left. Below the target face are three cartoon faces (frontal views), one of which is the same identity as the target face. The participant is asked to choose the face that looks the most like the target face. This task is loosely based on the Cambridge Face Perception Test (CFPT, Duchaine et al., 2007b), which involves sorting faces on a continuum from most to least like the target. Pilot testing of the CFPT with children indicated that children had difficulty with the concept of sorting images along a continuum, thus a 3-alternative forced choice method was adopted for the children's DFPT. Like in the CFPT, the target face and choice faces in the DFPT appear at different viewpoints to force reliance on typical face processing procedures by lessening the effectiveness of abnormal strategies such as feature matching (Hay and Young, 1982).

The test phase of the DFPT is identical to the practice, except that the eight target faces are male and female faces with neutral expressions chosen from the Dartmouth Database of Children's Faces (Dalrymple et al., 2013). Some of these target faces appear in the CFMT-K as distractors, but the two tasks use unique targets. Faces were converted to grayscale and cropped closely to remove hair and ears. Choice faces were created by morphing targets with a distractor face of the same gender. Each morph continuum progressed from the target identity to the distractor identity by increments of 10% (10% target/90% distractor, 20% target/80% distractor, etc.).

On each trial, a target face is presented at the top of the screen facing 30° to the viewer's left. Below the target are frontal views of three faces from that identity's morph continuum. Each choice face was made up of between 10% and 90% target. The greater the percent difference between the choice faces, the easier the trial, and the exact combination of choice faces was determined through extensive piloting. Each target appears 5 times with different combinations of choice faces from the morph continuum, for a total of 40 trials. The task is to choose the face that most resembles the target face. Participants respond by key press, and there is no time limit. Because the target and choice faces remain on the screen until a response is given, the memory demands of the task are minimal. Chance level performance for this test is 33.3%. Data from 92 typically developing children between 7 and 12 years of age indicates that this test has good internal consistency (Cronbach's *α* = 0.84).

### 1.2 Analysis

**Fig. 3:**
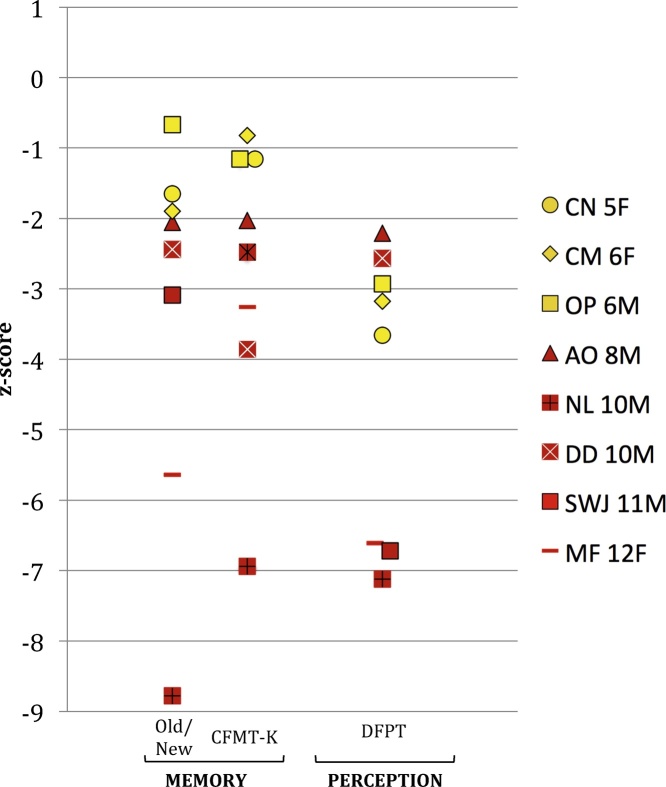
*Z*-scores for children with developmental prosopagnosia on tests of face memory (Old/New Faces, Cambridge Face Memory Test-Kids) and a test of face perception (Dartmouth Face Perception Test). Although the younger children (denoted in yellow) were not more than 2 standard deviations below the mean on the memory tests, their performance was near chance (see Fig. 2).

Each child's test scores were compared to means from at least 12 children of the same age, with the exception of CN (5-years-old), CM (6-years-old) and OP (6-years-old) whose scores were compared to data from 7-year-olds. We used two methods to compare the children's scores on each task to scores from age-matched control participants. First, we identified accuracy scores that were more than 2 standard deviations below the control mean. *Z*-scores are plotted in Fig. 3. We then ran Crawford and colleagues’ modified *t*-tests using SINGLIMS software (Crawford and Garthwaite, 2002, Crawford and Howell, 1998) to compare each child to their age-matched control group. This modified *t*-test is a more conservative measure of differences between single subjects and control groups with small sample sizes. All *t*-tests were two-tailed and *p*-values were compared to *α* = 0.05.

Floor effects in the youngest control group (i.e. 7-year-olds) made it difficult to detect scores that were more than 2 standard deviations below the mean. Therefore, although the three youngest DPs (CN, CM, and OP) performed near chance on the face memory tasks, their scores were not significantly below those of controls. For this reason, we will focus on the data from the five oldest DPs, but we provide data from the three younger DPs because they experience difficulties in daily life and their scores on face tests were extremely poor. In contrast, their IQ and object memory scores were relatively high (see Fig. 2), suggesting that they are capable of performing well on similar tests.

Note that it can be misleading to compare the magnitude of the *z*-scores of children of different ages because of the variability in control means and SDs as a function of age. For example, for the DFPT, the control mean and SD for 8-year-olds (*n* = 15) is *M* = 75.8%, SD = 16.2%, while the control mean and standard deviation for 12-year-olds (*n* = 14) is *M* = 89.9%, SD = 6.4%. Thus, the *z*-score for an 8-year-old who is at chance for this test will be −2.65, while the *z*-score for a 12-year-old who is similarly at chance on this test will be −8.86. For reference, the means and standard deviations from typically developing children that were used to calculate the z-scores are provided in Supplementary Table 1. Fig. 2 shows accuracy for individual DPs and may allow for more meaningful between subjects’ comparison.

### 1.3 Results

Face memory and face perception accuracy scores for all children with DP are presented in Supplementary Table 2 and Fig. 2, Fig. 3. *T*- and *p-*values from modified *t*-tests are also included in Supplementary Table 2. All of the five older children (AO, NL, DD, SWJ, and MF) were more than 2 standard deviations below the control mean on both face memory tests and the face perception test. For the most part, this was in line with results from the modified *t*-tests, which identified four of five children as scoring significantly below the control mean on the face memory tests, and all five children as scoring significantly below the control mean on the face perception test. AO's face memory scores were borderline (CFMT-K *p* = 0.062; Old/New *p* = 0.065).

The three younger children (CN, CM, and OP) were 2 standard deviations below the control mean on the Dartmouth Face Perception Test and the modified *t*-tests similarly classified these perception scores as being significantly different from the control group. Although the scores of the younger children were not significantly below control mean on the two tests of face memory, their scores were at, or near chance on these tasks: Chance is 33.3% for the CFMT-K (CN scored 37.5%; CM scored 43.8%; OP scored 37.5%) and 50.0% for the Old/New Faces task (CN scored 50.0%; CM scored 46.7%; OP scored 63.3%).

In contrast to the face memory scores, all children scored normally on the bicycle memory task, and only one (SWJ) scored in the impaired range on the Old/New Flowers task (see Supplementary Table 2). CN, CM, and OP had much higher accuracy for object memory than face memory suggesting that their low face memory scores were not a result of general cognitive factors. Although normal object recognition is not a requirement for a diagnosis of DP (some DPs have comorbid object recognition impairments, Duchaine and Nakayama, 2005), normal object memory scores provide evidence that participants understood the tasks and were capable of performing them.

## 2 Study 2: Adults

### 2.1 Method

#### 2.1.1 Participants

Adult DPs (*n* = 16, 11 females) and age-matched controls (*n* = 18, 11 females) were previously reported in a structural imaging study (Garrido et al., 2009). Like the children, these participants were recruited through faceblind.org. The mean age of the DPs was 31.5 years (SD = 7.4, range 20–46) and the mean age for controls was 28.9 (SD = 5.6, range 23–43). All participants reported being right handed.

#### 2.1.2 Assessment

**Fig. 4:**
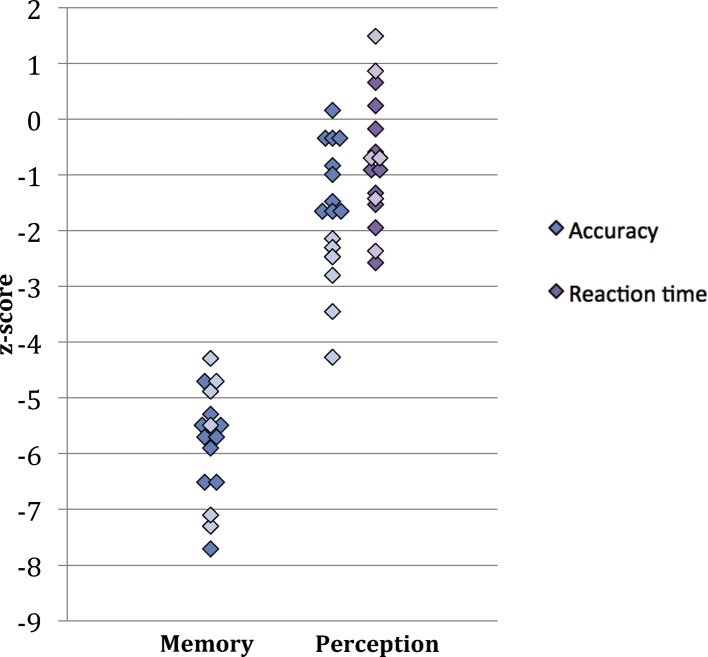
*Z*-scores for adults with developmental prosopagnosia on a test of face memory (Cambridge Face Memory Test) and a test of face perception (Cambridge Face Perception Test). Face memory scores plot accuracy, face perception scores plot accuracy and reaction time (RT). Z-scores for RT were multiplied by −1 so that slower than average performance is assigned a negative value. Darker colors represent individuals with normal CFPT accuracy scores; lighter colors represent individuals with CFPT accuracy scores that were >2 SD below the control mean.

All adult DPs were assessed at the Institute of Cognitive Neuroscience at University College London. Like the children, adult DPs took two tests of face memory (Cambridge Face Memory Test, Duchaine and Nakayama, 2006a; Old/New Faces, Duchaine and Nakayama, 2005), a test of face perception (Cambridge Face Perception Task, Duchaine et al., 2007b), and tests of object memory (Old/New Houses, Horses, and Cars). These tests are described below. Results from face tasks can be found in Table 1, Fig. 4, and Supplementary Table 3. Results from object tasks are in Supplementary Table 4. Low-level vision was assessed using the length, size, orientation, and position of gap subscales from the Birmingham Object Recognition Battery (BORB, Riddoch and Humphreys, 1993). These data were previously reported in Garrido et al. (2009), but to summarize, the DPs scored normally on all subscales, except DP13, who scored in the impaired range for length match only. This single score is not considered sufficient to suggest the presence of low-level visual impairment in this individual. DP17 was unavailable for BORB assessment.

**Table 1:** Data from adults with developmental prosopagnosia and age- and IQ-matched controls.

Participant info		Old/New Faces	CFMT	CFPT	
ID	Age/gender	(50%)	(33%)	(35%)	RT (s)
DP14	32 F	82.0*	56.9*	80.6	34
DP5	20 F	64.0*	44.4*	76.4	30
DP9	33 M	74.0*	51.4*	76.4	41
DP12	24 F	70.0*	50.0*	76.4	41
DP10	29 F	56.0*	36.1*	72.2	26
DP16	46 F	78.0*	52.8*	70.8	**51**
DP7	42 F	82.0*	51.4*	66.7	38
DP1	27 F	72.0*	50.0*	65.3	47
DP3	30 M	86.0*	48.6*	65.3	57*
DP8	43 M	88.0*	44.4*	65.3	45
DP6	35 M	82.0*	55.6*	**61.1**	39
DP13	25 F	58.0*	38.9*	59.7*	24
DP4	24 F	78.0*	51.4*	58.3*	18
DP17	36 M	70.0*	40.3*	55.6*	39
DP15	27 F	78.0*	56.9*	50.0*	46
DP2	31 F	78.0*	59.7*	43.1*	55*
Controls^a^	28.9 (5.7)	96.7 (3.4)	89.3 (6.9)	79.2 (8.5)	32 (9.6)

*Note*: Data were previously reported in Garrido et al. (2009); here it is sorted by CFPT scores. CFMT = Cambridge Face Memory Test; CFPT = Cambridge Face Perception Test. Chance level performance on these tests is indicated in parentheses. RTs are mean per trial. Bold indicates scores > 2SD above (RT) or below (accuracy) the control mean.

a Controls (*n* = 18, 11 females) means (SD).

* Scores significantly different from control group based on modified *t*-statistics (two-tailed, *α* = 0.05; Crawford and Garthwaite, 2002, Crawford and Howell, 1998).

Supplementary material related to this article can be found in the online version at http://dx.doi.org/10.1016/j.dcn.2014.07.003.

Table S3 Modified *t*-statistics (Crawford and Garthwaite, 2002, Crawford and Howell, 1998) for adults with developmental prosopagnosia compared to age-matched controls.

Table S4 Accuracy, and modified *t*-statistics (Crawford and Garthwaite, 2002, Crawford and Howell, 1998) for adults with developmental prosopagnosia on tests of object memory.

##### 2.1.2.1 Face memory tests

###### 2.1.2.1.1 Cambridge Face Memory Test

The Cambridge Face Memory Test (CFMT) is described in Duchaine and Nakayama (2006a). It has the same format as the Cambridge Face Memory Test-Kids with six target faces, but uses adult faces instead of children's faces.

*Old/New Faces* The Old/New Faces task is described in Duchaine and Nakayama (2005). Ten target and thirty non-target faces were chosen from yearbook photographs. All faces were female, grayscale, and cropped so that very little or no hair was visible. To achieve a standard pose, some of the images were flipped or rotated.

For the study portion of the task, the participant is presented with the 10 target items for 3 s per item. The 10 items are presented twice in the same order to improve encoding. During the test phase, the participant is presented with items one at a time and is asked to respond whether an item was a target item (old) or a non-target item (new) as quickly as possible with a mouse click. A total of 50 test items are presented consisting of 20 target items (10 targets × 2 presentations) and 30 non-targets (30 non-targets × 1 presentation). Chance level performance is 50%.

##### 2.1.2.2 Face perception test

###### 2.1.2.2.1 Cambridge Face Perception Test (CFPT)

The Cambridge Face Perception Test is described in detail in Duchaine et al. (2007b). It is a computerized sorting task in which participants arrange six facial images according to their similarity to a target face. The images were created by morphing six different individuals with each target face. The images contain 88%, 76%, 64%, 52%, 40%, and 28% of the target face. On each trial, the participant is presented with a 3/4 profile view of a target face above frontal views of six men's faces in a random order. The target face and sort faces appear at different viewpoints to lessen the effectiveness of feature matching (Hay and Young, 1982). The participant is given one minute to sort the images from most to least like the target face. Eight different sorts are presented both upright and inverted, with upright and inverted trials intermixed. One upright and one inverted practice trial is presented at the start of the test.

Scores for each item are computed by summing the deviations from the correct position for each face. For example, if a face is one position from its correct position, that is one error, if it is three positions away, that is three errors. Scores for the eight upright items and the eight inverted items are computed to determine total number of upright and inverted errors. The maximum number of errors on the eight trials is 144. Accuracy is computed by subtracting a participant's error score from the maximum number of errors and dividing this difference by the maximum number of errors (i.e. max errors − participant errors/max errors). Performance at chance is 35%.

#### 2.2 Analysis

Individuals in our adult prosopagnosia group were identified as being prosopagnosic in a previous report (Garrido et al., 2009). Like with the child DPs, we again used two methods to compare the adult scores to scores from the 18 IQ and age-matched control participants from Garrido et al. (2009). First, we identified CFMT, Old/New, and CFPT accuracy scores that were more than 2 standard deviations below the control mean, and CFPT reaction times that were more than 2 standard deviations above the control mean. We then used modified *t*-tests (Crawford and Garthwaite, 2002, Crawford and Howell, 1998) to compare each prosopagnosic to the control group on the same measures. All *t*-tests were two-tailed and *p*-values were compared to *α* = 0.05.

#### 2.3 Results

Scores from adult DPs and controls are presented in Table 1 and Fig. 4. *T*- and *p-*values from modified *t*-tests are in Supplementary Table 3. Again, the two methods of comparing DPs to controls were largely consistent: all DPs were more than 2 standard deviations below the control mean on the CFMT and Old/New Faces tasks, indicating impaired face memory. All CFMT and Old/New Faces scores were significantly below the control mean (all *p* < 0.025). In contrast, only 6 of the 16 DPs were more than 2 standard deviations below the control mean on the CFPT, indicating that at least 10 of the DPs scored normally on this measure of face perception. Using the modified *t*-tests, five of the 16 adult DPs scored significantly below the control mean on this task (DP6 was borderline: *p* = 0.054). Six of the adult DPs were within 1 SD of the control mean, and one scored above the control mean. We feel especially confident that these six DPs have normal facial identity perception because the performance of the control group was particularly good on this task compared to a previously reported control group: the mean for the control group used here was 79.2% (SD = 8.5) whereas the mean for the control group in Duchaine et al. (2007a) was 74.5% (SD = 8.5).

To confirm a dissociation between face memory and face perception in the 10 DPs who scored normally on the CFPT, we used Crawford and Garthwaite's (2007) Bayesian Standardized Difference Test to test whether, for each DP, the difference between scores on CFMT and CFPT was significantly larger than the mean difference between scores observed in controls. For all ten DPs, there was a significant difference between performance on the two tasks (all *p* < 0.004).

To determine whether normal accuracy on the CFPT could be accounted for by abnormally slow performance, we looked at the response times of the 10 DPs who scored in the normal range on this task. Two of these DPs had reaction times in that were more than 2 standard deviations above the mean and only one had a score that was significantly greater than the control group according to the modified *t*-tests (DP3: *p* = 0.021). Furthermore, as a group, the 10 DPs who scored in the normal range on the CFPT showed inversion effects (*M* = 22.4, SD = 7.5) that were comparable to those of the controls (*M* = 25.6, SD = 15.4), *t*(26) = 0.62, *p* = 0.538, and therefore indicative of the engagement of normal face processing procedures (Yin, 1969) rather than the use of feature matching strategies that do not depend on face processing.

## 3 Discussion

We measured the face perception and face memory abilities of eight children and 16 adults with developmental prosopagnosia (DP) to determine whether these components of face recognition are dissociable in DP. All of the children had impairments to both face perception and face memory, showing no evidence of a dissociation between these abilities. In contrast, at least half of the adults had face perception scores in the normal range, despite scoring in the impaired range on tests of face memory. The majority of these adults had normal reaction times and inversion effects on the face perception task, suggesting that they did not use alternative or abnormal strategies to achieve their normal accuracy scores. Thus, in contrast to the data from the children, the data from adults suggest that face perception and face memory are dissociable in DP and are consistent with data from adults with acquired prosopagnosia who have similarly shown two subtypes of the disorder, with some impaired at both face perception and face memory (Barton et al., 2004, Barton et al., 2002, Dalrymple et al., 2011, De Renzi et al., 1991) and others with normal face perception despite impairments of face memory (Barton et al., 2004, Barton et al., 2002, Dalrymple et al., 2011, De Renzi et al., 1991, Tippett et al., 2000). Taken together, our results suggest the proportion of DP kids with normal face perception may be lower than the proportion seen in adults with DP. Note though that although the findings raise the possibility that perceptual deficits are more common in DP children than adults, we expect children with normal facial identity perception will be identified in future work. Below we will first consider some basic methodological explanations for our results, but ultimately we will suggest that our findings may be best explained by more theoretical accounts of the data.

From a methodological standpoint, the difference between the child and adult DPs could be explained by a sampling bias. Our sample of children with DP was relatively small (*n* = 8) so we may have tested a biased sample of children by chance. If we assume the adult sample provides a representative distribution of DPs with normal and impaired facial identity perception, we can estimate the probability of testing a group eight DPs who all have impaired face perception. Six out of the 16 adult DPs (37.5%) had impaired face perception, making the probability of sampling eight other DPs who all have impaired face perception extremely small (*p* < 0.001). To be more conservative, we could classify normal performance as scores within one standard deviation of the mean (six of our adult DPs). In this case the probability of sampling eight new DPs with impaired perception remains small (*p* = 0.023). While these probabilities are rough estimates based on the present sample, they suggest it is unlikely that our finding that all eight children with DP had impaired face perception was due to chance.

A second methodological explanation for our data is that a systematic factor affected which participants came to the attention of our lab. Our sample of children was drawn from a list of children whose parents contacted us because they believe their child has face recognition deficits. It is possible that the combination of impaired face perception and face memory is more noticeable in daily life than face memory impairments alone and that we were therefore only contacted by parents whose children are impaired with both aspects of face recognition. However, the adults with DP were also self-selected, meaning that this argument would be expected to apply to both samples. That is, if the combination of face perception and face memory deficits is indeed more noticeable in daily life than face memory deficits alone, then we would expect our adult sample to contain a higher proportion of individuals who experience both deficits than those who have normal face perception with impaired face memory. Instead we found that adult DPs with perceptual deficits made up less than half of our adult sample. Moreover, it is unclear why deficits with perception and memory would be more noticeable in daily life than deficits with face memory alone.

An additional methodological explanation for our data is that the perceptual tests used with children and adults measure different abilities. Both tests require comparison of a target face to morphed test faces shown from different views, but the tests did differ in the number of test faces presented and the responses required. Children chose which one of three faces looked most like the target face whereas adults sorted six faces in terms of similarity to the target face. The reason for the difference in methodology is that children, particularly the younger ones, had difficulties with the concept of sorting on a continuum. It is possible that the different tests engage very different perceptual processes, but given the similarity of the tests, we believe that is unlikely.

Methodological considerations aside, we believe that these data may provide new insights into the developmental trajectory of DP. Specifically, our data raise the following question: how can a single subtype of child DPs (i.e. all showing impairments of face perception and face memory) develop into two subtypes of adult DPs (i.e. those who are impaired at both face perception and face memory, versus those who are impaired at face memory alone)?

One answer to this question is that face perception can improve over time in some children with DP. Although Weigelt et al. (2014) reported that normal face perception follows the same developmental trajectory as object perception, it is possible that children with DP can show delayed development of face perception, while their face memory remains poor into adulthood. In other words, children with DP might “outgrow” their face perception deficits. This would suggest that a subset of our child sample could show improved scores on our perception tasks later in life. Longitudinal work is needed to test this possibility.

An alternative, though not mutually exclusive, answer to the question posed above is that we are failing to detect children whose deficits are restricted to face memory alone. This explanation is related to Weigelt et al.’s (2014) finding that normal face memory is slow to develop relative to memory for other classes of objects. Specifically, it is possible that in childhood, some individuals have normal face perception, and poor, *but not impaired*, face memory relative to their peers (i.e. because their peers also have relatively poor face memory). Yet as their peers show improvements for face memory with age, these individuals may continue to struggle with face memory, at which point they would be measurably impaired. Indeed, parents of some children we have tested have provided anecdotal evidence that their child has face recognition difficulties, but upon testing, their child performed within the normal range for both face perception and face memory. Testing the possibility of later emergence of face memory impairments would require more sensitive measures of face memory for younger children (i.e. to identify memory impairments at a younger age), or longitudinal follow-up with children who anecdotally struggle with face recognition in daily life, yet perform in the low but normal range our tests of face perception and memory.

In addition to raising questions about the developmental trajectory of DP, our results have implications for our understanding of DP, and the development of condition-specific treatment for children and adults with face recognition deficits. The finding that perception is impaired in all cases of childhood DP, but only in half of the adults with DP, suggests the possibility that face perception can improve or recover prior to, or during, adulthood. Moreover, we believe it is generally assumed that children with DP inevitably become adults with DP, but our findings raise the question of whether *both* face perception and face memory can improve (i.e. that DP can resolve itself over time). Longitudinal work with children with DP is needed to test these possibilities. With regards to treatment, our results suggest that training strategies should target both face perception and face memory in children with DP. In contrast, treatment strategies for adults should vary according to individual needs: those with perceptual impairments should receive training targeted at face perception and face memory, while those with normal face perception should focus on improving their memory for faces. Ultimately, targeting individual needs should lead to more positive treatment outcomes.

## Conflict of interest statement

The authors have no conflict of interest to report.

## Footnotes

1 To reflect the division between deficits of face perception and face memory, some have reconditioned terms from the literature on visual agnosia (Lissauer, 1890), defining an “apperceptive” type of prosopagnosia as an inability to analyze the perceptual elements of a face, and contrasting it with an “associative” type of prosopagnosia, defined as the inability to form or access facial memories, despite intact face perception (Barton, 2003, Barton et al., 2002, Damasio et al., 1990, Davies-Thompson et al., 2014, De Renzi et al., 1991, Fox et al., 2008, McConachie, 1976).
