# Supplementary material for: Dissociation between face perception and face memory in adults, but not children, with developmental prosopagnosia
Source: Dev Cogn Neurosci. 2014 Aug 1;10:10–20. doi: 10.1016/j.dcn.2014.07.003 (PMC6987906; doi:10.1016/j.dcn.2014.07.003)
Supplement: Table S1 — Mean scores (%) by age for typically developing children on tests of face memory, face perception, and object memory. [file mmc1.docx]

**Supplementary Table 1.** *Mean scores (%) by age for typically developing children on tests of face memory, face perception, and object memory*

|  |  | Age (years) | | | | | |
| --- | --- | --- | --- | --- | --- | --- | --- |
| Test | Chance | 7 | 8 | 9 | 10 | 11 | 12 |
|  |  |  |  |  |  |  |  |
| Face Memory |  |  |  |  |  |  |  |
|  |  |  |  |  |  |  |  |
| CFMT-K^a^ | 33% | 59.0(18.5)  n=15 | 70.2 (16.1)  n=20 | 80.2 (12.0)  n=16 | 84.7 (7.2)  n=15 | 78.4 (13.7)  n=15 | 79.4 (8.6)  n=14 |
|  |  |  |  |  |  |  |  |
| Old New Faces | 50% | 72.3 (13.5)  n=15 | 77.8 (10.2)  n=15 | 81.5 (11.0)  n=17 | 88.7 (6.3)  n=15 | 87.0 (8.8)  n=16 | 90.0 (5.9)  n=15 |
| Face Perception |  |  |  |  |  |  |  |
|  |  |  |  |  |  |  |  |
| DFPT | 33% | 72.7 (10.3)  n=14 | 75.8 (16.2)  n=15 | 82.2 (13.3)  n=15 | 84.8 (7.7)  n=15 | 88.1 (7.9)  n=16 | 89.8 (6.4)  n=14 |
|  |  |  |  |  |  |  |  |
| Object memory |  |  |  |  |  |  |  |
|  |  |  |  |  |  |  |  |
| CBMT^a^ | 33% | 69.1 (15.6)  n=12 | 82.1 (9.3)  n=12 | 79.9 (14.8)  n=15 | 68.0 (9.4)  n=15 | 71.6 (9.6)  n=15 | 75.6 (8.7)  n=16 |
|  |  |  |  |  |  |  |  |
| Old-New Flowers | 50% | 81.7 (9.9)  n=14 | 85.0 (8.2)  n=16 | 90.2 (10.3)  n=14 | 88.4 (8.0)  n=15 | 89.6 (6.9)  n=15 | 90.5 (8.6)  n=15 |
|  |  |  |  |  |  |  |  |

*Note: CFMT-K = Cambridge Face Memory Test - Kids; DFPT = Dartmouth Face Perception Test; CBMT = Cambridge Bicycle Memory Test. Standard deviation in parentheses.*

^a^ For the CFMT-K and CBMT children aged 7-9 years memorized 4 targets, children aged 10-12 years memorized 6 targets.
