# Supplementary material for: Dissociation between face perception and face memory in adults, but not children, with developmental prosopagnosia
Source: Dev Cogn Neurosci. 2014 Aug 1;10:10–20. doi: 10.1016/j.dcn.2014.07.003 (PMC6987906; doi:10.1016/j.dcn.2014.07.003)
Supplement: Table S2 — Accuracy, and modified t-statistics (Crawford and Garthwaite, 2002, Crawford and Howell, 1998) for children with developmental prosopagnosia on subscales of the Birmingham Object Recognition Battery (BORB, Riddoch and Humphreys, 1993), tests of face and object memory, and face perception. [file mmc2.docx]

**Supplementary Table 2.** *Accuracy, and modified t-statistics (Crawford & Garthwaite, 2002; Crawford & Howell, 1998) for children with developmental prosopagnosia on subscales of the Birmingham Object Recognition Battery (BORB, Riddoch & Humphreys, 1993), tests of face and object memory, and face perception*

|  | | Child (age/gender) | | | | | | | |
| --- | --- | --- | --- | --- | --- | --- | --- | --- | --- |
| Test | Chance | CN (5F) | CM (6F) | OP (6M) | AO (8M) | DD (10M) | NL (10M) | SWJ (11M) | MF (12F) |
| WASI-II |  |  |  |  |  |  |  |  |  |
| VIQ | N/A | 138 ^a^ | 122 | N/A | 132 | 113 | 120 | 154 | 91 |
| PIQ | N/A | 120 ^a^ | 112 | N/A | 122 | 105 | 117 | 126 | 86 |
| Low-level vision (BORB) |  |  |  |  |  |  |  |  |  |
| Length | 50% | 80.0  t(38)=-1.79  *p=*0.081 | 83.3  t(38)=-1.17  *p=*0.248 | N/A | 93.3  t(38)=0.68  *p=*0.501 | 83.3  t(38)=-1.17  *p=*0.248 | 96.7  t(38)=1.30  *p=*0.203 | N/A | 83.3  t(38)=-1.17  *p=*0.248 |
|  |  |  |  |  |  |  |  |  |  |
| Size | 50% | 76.7  t(38)=-1.77  *p=*0.085 | 76.7  t(38)=-1.77  *p=*0.085 | N/A | 90.0  t(38)=-0.12  *p=*0.902 | 83.3  t(38)=-0.95  *p=*0.350 | 90.0  t(38)=-0.12  *p=*0.902 | N/A | 86.7  t(38)=-0.54  *p=*0.596 |
|  |  |  |  |  |  |  |  |  |  |
| Orientation | 50% | 70.0  t(38)=-1.44  *p=*0.157 | 70.0  t(38)=-1.44  *p=*0.157 | N/A | 96.7  t(38)=1.60  *p=*0.119 | 80.0  t(38)=-0.30  *p=*0.763 | 76.7  t(38)=-0.68  *p=*0.498 | N/A | 83.3  t(38)=0.08  *p=*0.940 |
|  |  |  |  |  |  |  |  |  |  |
| Position | 50% | **62.5***  **t(37)=-2.49**  ***p=*0.017** | 87.5  t(37)=-0.03  *p=*0.980 | N/A | 87.5  t(37)=-0.03  *p=*0.980 | 77.5  t(37)=-1.01  *p=*0.318 | 92.5  t(37)=0.47  *p=*0.642 | N/A | 75.0  t(37)=-1.26  *p=*0.216 |
| Face Memory |  |  |  |  |  |  |  |  |  |
| CFMT-K^b^ | 33% | 37.5  t(14)=-1.13  *p=*0.279 | 43.8  t(14)=-0.80  *p=*0.440 | 37.5  t(14)=-1.13  *p=*0.279 | **37.5**  **t(19)=-1.98**  ***p=*0.062** | **56.9***  **t(14)=-3.74***  ***p=*0.002** | **34.7***  **t(14)=-6.72***  ***p<*0.001** | **44.4***  **t(14)=-2.40***  ***p=*0.031** | **51.4***  **t(13)=-3.15***  ***p=*0.008** |
| Old New Faces | 50% | 50.0  t(14)=-1.60  *p=*0.132 | 46.7  t(14)=-1.84  *p=*0.088 | 63.3  t(14)=-0.65  *p=*0.529 | **56.7**  **t(14)=-2.00**  ***p=*0.065** | **73.3***  **t(14)=-2.37***  ***p=*0.033** | **33.3***  **t(14)=-8.51***  ***p<*0.001** | **60.0***  **t(15)=-2.98***  ***p=*0.009** | **56.7***  **t(14)=-5.47***  ***p<*0.001** |
| Face Perception |  |  |  |  |  |  |  |  |  |
| DFPT | 33% | **35.0***  **t(13)=-3.54***  ***p=*0.004** | **40.0***  **t(13)=-3.07***  ***p=*0.009** | **42.5***  **t(13)=-2.83***  ***p=*0.014** | **40.0***  **t(14)=-2.14***  ***p=*0.050** | **65.0***  **t(14)=-2.49***  ***p=*0.026** | **30.0***  **t(14)=-6.89***  ***p<*0.001** | **35.0***  **t(15)=-6.52***  ***p<*0.001** | **47.5***  **t(13)=-6.40***  ***p<*0.001** |
| Object Memory |  |  |  |  |  |  |  |  |  |
| CBMT^b^ | 33% | 77.1  t(11)=0.49  *p=*0.632 | 50.0  t(11)=-1.18  *p=*0.264 | N/A | 79.2  t(11)=-0.30  *p=*0.770 | 68.1  t(14)=0.01  *p=*0.992 | 84.7  t(14)=1.72  *p=*0.107 | 59.7  t(14)=-1.20  *p=*0.250 | 72.2  t(15)=-0.38  *p=*0.710 |
| Old New Flowers | 50% | 70.0  t(13)=-1.14  *p=*0.274 | 83.3  t(13)=0.16  *p=*0.878 | 80.0  t(13)=-0.17  *p=*0.871 | 90.0  t(15)=0.59  *p=*0.563 | 76.7  t(14)=-1.42  *p=*0.179 | 90.0  t(14)=0.19  *p=*0.849 | **73.3***  **t(14)=-2.29***  ***p=*0.038** | 86.7  t(14)=-0.43  *p=*0.675 |
|  |  |  |  |  |  |  |  |  |  |

*Note: WASI-II = Wechsler Abbreviated Scale of Intelligence – II (Wechsler, 2011); CFMT-K = Cambridge Face Memory Test - Kids; DFPT= Dartmouth Face Perception Test; CBMT = Cambridge Bicycle Memory Test. Bold indicates scores > 2SD below the control mean. * indicates scores significantly different than age-matched control group based on modified t-tests (two-tailed t-test, α=0.05). Scores for CN (5F), CM (6F), and OP (6M) were compared to scores from 7-year-olds. VIQ=Verbal IQ; PIQ=Performance IQ, N/A=Not available.*

**^a^** IQ scores for CN (5 years, 10 months, 20 days at the time of assessment), were calculated based on norms from 6-years-olds.

**^b^** For the CFMT-K and CBMT children aged 7-9 years memorized 4 targets, children aged 10-12 years memorized 6 targets.
