# Supplementary material for: Dissociation between face perception and face memory in adults, but not children, with developmental prosopagnosia
Source: Dev Cogn Neurosci. 2014 Aug 1;10:10–20. doi: 10.1016/j.dcn.2014.07.003 (PMC6987906; doi:10.1016/j.dcn.2014.07.003)
Supplement: Table S3 — Modified t-statistics (Crawford and Garthwaite, 2002, Crawford and Howell, 1998) for adults with developmental prosopagnosia compared to age-matched controls. [file mmc3.docx]

**Supplementary Table 3.** *Modified t-statistics (Crawford & Garthwaite, 2002; Crawford & Howell, 1998) for adults with developmental prosopagnosia compared to age-matched controls*

| Participant info | | Old/New Faces | CFMT | CFPT | |
| --- | --- | --- | --- | --- | --- |
|  | |  |  |  | |
| ID | Age/Gender | (50%) | (33%) | (35%) | RT (s) |
|  |  |  |  |  |  |
| DP14 | 32 F | **t(17)=-4.21***  ***p*=0.001** | **t(17)=-4.57***  ***p*<0.001** | t(17)=0.16  *p*=0.875 | t(17)=0.20  *p*=0.842 |
|  |  |  |  |  |  |
| DP5 | 20 F | **t(17)=-9.36***  ***p*<0.001** | **t(17)=-6.33***  ***p*<0.001** | t(17)=-0.32  *p*=0.752 | t(17)=-0.20  *p*=0.842 |
|  |  |  |  |  |  |
| DP9 | 33 M | **t(17)=-6.50***  ***p*<0.001** | **t(17)=-5.35***  ***p*<0.001** | t(17)=-0.32  *p*=0.752 | t(17)=0.91  *p*=0.374 |
|  |  |  |  |  |  |
| DP12 | 24 F | **t(17)=-7.64***  ***p*<0.001** | **t(17)=-5.54***  ***p*<0.001** | t(17)=-0.32  *p*=0.752 | t(17)=0.91  *p*=0.374 |
|  |  |  |  |  |  |
| DP10 | 29 F | **t(17)=-11.65***  ***p*<0.001** | **t(17)=-7.51***  ***p*<0.001** | t(17)=-0.80  *p*=0.434 | t(17)=-0.61  *p*=0.551 |
|  |  |  |  |  |  |
| DP16 | 46 F | **t(17)=-5.35***  ***p*<0.001** | **t(17)=-5.15***  ***p*<0.001** | t(17)=-0.96  *p*=0.350 | **t(17)=1.93**  ***p*=0.071** |
|  |  |  |  |  |  |
| DP7 | 42 F | **t(17)=-4.21***  ***p*=0.001** | **t(17)=-5.35***  ***p*<0.001** | t(17)=-1.43  *p*=0.170 | t(17)=0.61  *p*=0.551 |
|  |  |  |  |  |  |
| DP1 | 27 F | **t(17)=-7.07***  ***p*<0.001** | **t(17)=-5.54***  ***p*<0.001** | t(17)=-1.59  *p*=0.130 | t(17)=1.52  *p*=0.147 |
|  |  |  |  |  |  |
| DP3 | 30 M | **t(17)=-3.06***  ***p*=0.007** | **t(17)=-5.74***  ***p*<0.001** | t(17)=-1.59  *p*=0.130 | **t(17)=2.54***  ***p*=0.021** |
|  |  |  |  |  |  |
| DP8 | 43 M | **t(17)=-2.49***  ***p*=0.023** | **t(17)=-6.33***  ***p*<0.001** | t(17)=-1.59  *p*=0.130 | t(17)=1.32  *p*=0.205 |
|  |  |  |  |  |  |
| DP6 | 35 M | **t(17)=-4.21***  ***p*=0.001** | **t(17)=-4.75***  ***p*<0.001** | **t(17)=-2.07**  ***p*=0.054** | t(17)=0.71  *p*=0.488 |
|  |  |  |  |  |  |
| DP13 | 25 F | **t(17)=-11.08***  ***p*<0.001** | **t(17)=-7.11***  ***p*<0.001** | **t(17)=-2.23***  ***p*=0.039** | t(17)=-0.81  *p*=0.429 |
|  |  |  |  |  |  |
| DP4 | 24 F | **t(17)=-5.35***  ***p*<0.001** | **t(17)=-5.35***  ***p*<0.001** | **t(17)=-2.39***  ***p*=0.029** | t(17)=-1.42  *p*=0.174 |
|  |  |  |  |  |  |
| DP17 | 36 M | **t(17)=-7.64***  ***p*<0.001** | **t(17)=-6.91***  ***p*<0.001** | **t(17)=-2.70***  ***p*=0.015** | t(17)=0.71  *p*=0.488 |
|  |  |  |  |  |  |
| DP15 | 27 F | **t(17)=-5.35***  ***p*<0.001** | **t(17)=-4.57***  ***p*<0.001** | **t(17)=-3.34***  ***p*=0.004** | t(17)=1.42  *p*=0.174 |
|  |  |  |  |  |  |
| DP2 | 31 F | **t(17)=-5.35***  ***p*<0.001** | **t(17)=-4.18***  ***p*=0.001** | **t(17)=-4.13***  ***p*=0.001*** | **t(17)=2.33***  ***p*=0.032** |

*Note: Data was previously reported in Garrido et al. (2009); here it is sorted by CFPT scores. CFMT = Cambridge Face Memory Test; CFPT = Cambridge Face Perception Test. Chance level performance on these tests is indicated in parentheses. RTs are mean per trial for upright faces. Bold indicates scores > 2SD above (RT) or below (accuracy) the control mean. * indicates scores significantly different from control group based on modified t-statistics (two-tailed, α=0.05).*
