# Supplementary material for: Dissociation between face perception and face memory in adults, but not children, with developmental prosopagnosia
Source: Dev Cogn Neurosci. 2014 Aug 1;10:10–20. doi: 10.1016/j.dcn.2014.07.003 (PMC6987906; doi:10.1016/j.dcn.2014.07.003)
Supplement: Table S4 — Accuracy, and modified t-statistics (Crawford and Garthwaite, 2002, Crawford and Howell, 1998) for adults with developmental prosopagnosia on tests of object memory. [file mmc4.docx]

**Supplementary Table 4.** *Accuracy, and modified t-statistics (Crawford & Garthwaite, 2002; Crawford & Howell, 1998) for adults with developmental prosopagnosia on tests of object memory*

| Participant info | | Old/New Houses | Old/New Horses | Old/New Cars |
| --- | --- | --- | --- | --- |
|  | |  |  |  |
| ID | Age/Gender | (50%) | (50%) | (50%) |
|  |  |  |  |  |
| DP14 | 32 F | 44  t(17)=-0.43, *p*=0.671 | 41  t(17)=-1.32, *p*=0.205 | **40**  **t(17)=-1.99, *p*=0.063*** |
|  |  |  |  |  |
| DP5 | 20 F | 49  t(17)=1.07, *p*=0.300 | 46  t(17)=-0.04, *p*=0.966 | 43  t(17)=-0.94, *p*=0.361 |
|  |  |  |  |  |
| DP9 | 33 M | 43  t(17)=-0.73, *p*=0.474 | 41  t(17)=-1.32, *p*=0.205 | 46  t(17)=0.12, *p*=0.909 |
|  |  |  |  |  |
| DP12 | 24 F | 44  t(17)=-0.43, *p*=0.671 | 46  t(17)=-0.04, *p*=0.966 | 45  t(17)=-0.24, *p*=0.817 |
|  |  |  |  |  |
| DP10 | 29 F | 46  t(17)=0.17, *p*=0.868 | 49  t(17)=0.72, *p*=0.481 | 44  t(17)=-0.59, *p*=0.565 |
|  |  |  |  |  |
| DP16 | 46 F | 45  t(17)=-0.13, *p*=0.896 | 42  t(17)=-1.06, *p*=0.303 | **40**  **t(17)=-1.99, *p*=0.063*** |
|  |  |  |  |  |
| DP7 | 42 F | 45  t(17)=-0.13, *p*=0.896 | 41  t(17)=-1.32, *p*=0.205 | **37**  **t(17)=-3.05, *p*=0.007*** |
|  |  |  |  |  |
| DP1 | 27 F | 41  t(17)=-1.33 *p*=0.200 | **31**  **t(17)=-3.87, *p*=0.001*** | **31**  **t(17)=-5.16, *p*<0.001*** |
|  |  |  |  |  |
| DP3 | 30 M | 45  t(17)=-0.13, *p*=0.896 | 45  t(17)=-0.30, *p*=0.769 | 41  t(17)=-1.64, *p*=0.119 |
|  |  |  |  |  |
| DP8 | 43 M | 41  t(17)=-1.33 *p*=0.200 | 40  t(17)=-1.57, *p*=0.134 | **39**  **t(17)=-2.34, p=0.032*** |
|  |  |  |  |  |
| DP6 | 35 M | 41  t(17)=-1.33 *p*=0.200 | 40  t(17)=-1.57, *p*=0.134 | 48  t(17)=0.82, *p*=0.424 |
|  |  |  |  |  |
| DP13 | 25 F | 45  t(17)=-0.13, *p*=0.896 | 41  t(17)=-1.32, *p*=0.205 | **38**  **t(17)=-2.70, *p*=0.015*** |
|  |  |  |  |  |
| DP4 | 24 F | 42  t(17)=-1.03, *p*=0.316 | 47  t(17)=0.21, *p*=0.835 | 43  t(17)=-0.94, p=0.361 |
|  |  |  |  |  |
| DP17 | 36 M | 46  t(17)=0.17, *p*=0.868 | 43  t(17)=-0.81, *p*=0.430 | **40**  **t(17)=-1.99, *p*=0.063*** |
|  |  |  |  |  |
| DP15 | 27 F | 43  t(17)=-0.73, *p*=0.474 | 40  t(17)=-1.57, *p*=0.134 | **39**  **t(17)=-2.34, p=0.032*** |
|  |  |  |  |  |
| DP2 | 31 F | 39  t(17)=-1.94, *p*=0.070 | 39  t(17)=-1.83, *p*=0.085 | **31**  **t(17)=-5.16, *p*<0.001*** |
|  |  |  |  |  |
| Controls **^a^** | 28.9 (5.7) | 45.4 (3.24) | 46.2 (3.82) | 45.7 (2.77) |

*Note: Data was previously reported in Garrido et al. (2009). Chance level performance on these tests is indicated in parentheses. Bold indicates scores > 2SD below the control mean. * indicates scores significantly different from control group based on modified t-statistics (two-tailed, α=0.05).*

**^a^** Controls (n=18, 11 females) means (SD)

References

Crawford, J., & Garthwaite, P. (2002). Investigation of the single case in neuropsychology: Confidence limits on the abnormality of test scores and test score differences. Neuropsychologia, 40, 1196-1208.

Crawford, J., & Howell, D. (1998). Comparing an individual's test score against norms derived from small samples. The Clinical Neuropsychologist, 12(4), 482-486.

Garrido, L., Furl, N., Draganski, B., Weiskopf, N., Stevens, J., Tan, G., . . . Duchaine, B. (2009). Voxel-based morphometry reveals reduced grey matter volume in the temporal cortex of developmental prosopagnosics. Brain, 132, 3443-3455.

Riddoch, J., & Humphreys, G. (1993). BORB: Birmingham object recognition battery. Hove, UK: Erlbaum.

Wechsler, D. (2011). Wechsler Abbreviated Scale of Intelligence (WASI-II). USA: Pearson.
